# Flavan-3-ols Content in Red Raspberry Leaves Increases under Blue Led-Light Irradiation

**DOI:** 10.3390/metabo9030056

**Published:** 2019-03-21

**Authors:** Ryo Kobori, Seiya Hashimoto, Hayato Koshimizu, Shuich Yakami, Mizuki Hirai, Kenta Noro, Takashi Kawasaki, Akiko Saito

**Affiliations:** 1 Graduate School of Engineering, Osaka Electro-Communication University (OECU), 18-8 Hatsu-cho, Neyagawa-shi, Osaka 572-8530, Japan; me17a002@oecu.jp (R.K.); me16a013@oecu.jp (S.Y.); de18a001@oecu.jp (M.H.); me15a005@oecu.jp (K.N.); 2 Faculty of Engineering, Osaka Electro-Communication University (OECU), 18-8 Hatsu-cho, Neyagawa-shi, Osaka 572-8530, Japan; eu15a079@oecu.jp (S.H.); eu15a036@oecu.jp (H.K.); 3 Research Institute for Sustainable Humanosphere, Kyoto University, Uji, Kyoto 611-0011, Japan; kawasaki.takashi.2x@kyoto-u.ac.jp

**Keywords:** polyphenols, light, temperature, controlled environment, biosynthesis, leaf extracts, HPLC, structure-function relations

## Abstract

Berry fruits are well known to contain large amounts of polyphenol compounds. Among them, flavan-3-ol derivatives are a group of secondary metabolism compounds currently attracting a great deal of attention owing to their health benefits. Not only the fruits, but also the leaves of raspberry plants, are highly esteemed for tea making around the world and are largely used for food. In this report, we discuss the results of our study on the effect of light and temperature on polyphenol accumulation in raspberry leaves. When raspberry was cultivated in a plant factory unit and light intensity, wavelength, and temperature were varied, the amount of total polyphenol increased under blue light. Quantitative determination of (+)-catechin, (–)-epicatechin, procyanidin B4, flavan-3-ol trimer, which are flavan-3-ol derivatives, was carried out using HPLC, whereby we confirmed their increase under blue light. Semi-quantitative RT-PCR showed correlation between chalcone synthase (CHS) gene expression and the amounts of the compounds measured in the leaves.

## 1. Introduction

There is currently great interest in the investigation of compounds from food sources with biological activities, as they are generally considered highly safe because they are consumed as part of the general daily diet. In particular, polyphenols contained in many health foods, as well as in vegetables and fruits, are thought to have various health benefits [1,2]. Among them, flavan-3-ol derivatives are general plant-derived physiologically active compounds known to be highly functional. In particular, the main ingredient of green tea-polyphenols shows various moderate bioactivities without severe toxicity and its health promoting effects have been extensively studied. Although various biologically active flavan-3-ol derivatives are present as minor constituents in plants as well as in green tea, their biological activities have yet to be revealed, mainly owing to their relative unavailability. Therefore, the development of new methods to elucidate the biosynthesis pathway, polymerization mechanisms, and transport of flavan-3-ol derivatives has become an important and active field of research [3,4]. Especially, developing a method to control the biosynthesis of flavan-3-ol derivatives with strong biological activities in plants would be a tool of great value to produce highly functional plants and foods.

The production of polyphenolic compounds is affected by light irradiation, and it is thought that this can be used to increase the amount of polyphenol secondary metabolites, including flavan-3-ol derivatives. For example, short-term ultraviolet C irradiation was shown to stimulate biosynthetic pathways, leading to enhancement of the accumulation of polyphenol compounds [5]. Similarly, long-term irradiation of visible blue light showed a similar effect on the amounts of flavonoids produced in Chinese cabbage [6]. These reports demonstrate the possibility of controlling the amounts of specific polyphenol compounds in plants, including flavan-3-ol derivatives; furthermore, they indicate that environmental conditions has a large influence on polyphenol production, which causes difficulty in the functional evaluation of plant extracts.

Flavan-3-ol derivatives are thought to promote health because of their various functionalities. However, their functional evaluation and the elucidation of the mechanism of action involved require the availability of these compounds in quantities large enough to enable the conduction of the structure-activity relationship (SAR) studies needed. Therefore, we developed the stereoselective synthesis method for producing flavan-3-ol derivatives, oligomeric compounds [7,8,9,10,11,12], galloyled derivatives [13,14,15,16,17], and acylated derivatives [18,19,20] for SAR studies, such as anti-oxidative activity [9,13], radical scavenging activity [14,15], Maillard reaction-inhibitory activity, [9] DNA polymerase inhibitory activity [10,13,14,15,18], HeLa S3 proliferation-inhibitory activity [11,16,17,20], and anti-viral activity [21]. These studies showed that fine structural differences, such as differences in stereochemistry and presence or absence of modifications, are important to demonstrate biological activity. In addition, these organic synthesis studies allowed us to store various standard compounds which are not commercially available and to construct a flavan-3-ol derivative library for chemical analysis studies and SAR studies which, overall, have revealed that among polyphenols, flavan-3-ol derivatives possess high functionality and that oligomers exhibit various functionalities.

Our next challenge was to achieve the effective, selective production of high-functional flavan-3-ol derivatives in plants. Thus, we selected red raspberry (*Rubus idaeus* L.) as our experimental model plant because it is a species widely known for its edible fruits and as polyphenol rich and highly beneficial for human health [22,23]. Furthermore, they are most commonly known as food products and their extracts are also known as anti-inflammatory [24] and antimicrobial properties [25]. As mentioned above, numerous studies report increasing polyphenol content in various plants by light irradiation management. Therefore, we planned studies to irradiate raspberries with LED light to increase polyphenols, especially flavan-3-ol compounds.

**Figure 1:**
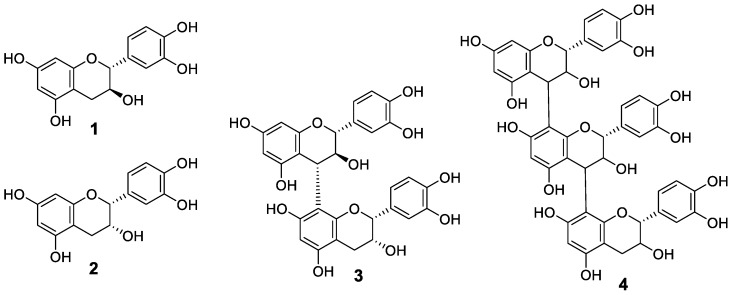
The structures of flavan-3-ol derivatives contained in raspberry.

In this report, we discuss the method for raspberry cultivation in a plant factory unit kept under constant environmental conditions, and the response in polyphenol production in the leaves to light wavelength. When blue LED light was used, the amounts of total polyphenols and flavan-3-ol derivatives (Figure 1), (+)-catechin (**1**), (–)-epicatechin (**2**), procyanidin B4 (**3**), and flavan-3-ol trimer (**4**), were all increased. As a result of PCR analysis to clarify the reason for the increase, it was possible to confirm the correlation between the expression of the related gene *CHS* and the increase in the compound level.

## 2. Results and Discussion

### 2.1. Raspberry Cultivation in a Plant Factory Unit and LED Light Irradiation

Various polyphenol compounds, such as anthocyanin, flavan-3-ol derivatives, including proanthocyanidins, quercetin derivatives, and hydrolyzed tannin, are contained in both fruits and leaves of red raspberries [26,27]. Among these polyphenol compounds, we were interested in flavan-3-ol derivatives with high functionality and aimed to determine cultivation conditions in which their content increased. However, in general, the amount of secondary metabolites in plants varies greatly, depending on cultivation environment. In a study on outdoor cultivation of raspberries, fluctuations in anthocyanin content were evaluated seasonally for two years, and they were observed to vary greatly [28]. Therefore, to evaluate the fluctuation in the amount of each polyphenol compound, we cultivated red raspberries in a plant factory unit under a controlled environment. Cultivation in sand was selected, as healthy tomato and strawberry plants can be easily grown in sand, being an inorganic growth medium, provides for much more controllable conditions than organic substrates [25].

Recently, numerous studies have reported increased amounts of functional compounds by applying various light wavelengths. Thus, for example, polyphenols reportedly increase upon UV light exposure shortly before [29] or after harvest [5]. However, UV light is high-energy radiation that can potentially hurt the people working in the area. Therefore, we used visible light for our experimental purposes. In basil callus cultures, polyphenols such as rosmarinic acid increased under blue LED irradiation, while anthocyanidins increased with red LED irradiation [30]. In red light irradiation, it is possible that flavan-3-ol derivatives are consumed and anthocyanidins are increased. Therefore, in this study, blue LED irradiation was used.

Table 1 shows the raspberry (Himbo Top) cultivation conditions in the plant factory unit and the dry weight of the methanol extracts obtained from 1.0 g samples of dried leaves. Conditions A and B consisted in commercially available white LED light; conditions C to G varied by changing the proportion of blue and red LED lights. Cultivation under condition H consisted in no LED light provision. Each cultivation condition was maintained for seven days, after which the leaves were harvested for extraction. Cultivation conditions did not largely affect the extracted weight obtained; however, when plants were cultivated under blue light only, the extract tended to be less. Each extract was adjusted to 100 mg/mL solution with dimethyl sulfoxide (DMSO) and used for quantitative analysis and radical scavenging activity.

### 2.2. Analysis of Total Polyphenols and Total Proanthocyanidin Amounts in Extracts from Raspberry Leaves

**Figure 2:**
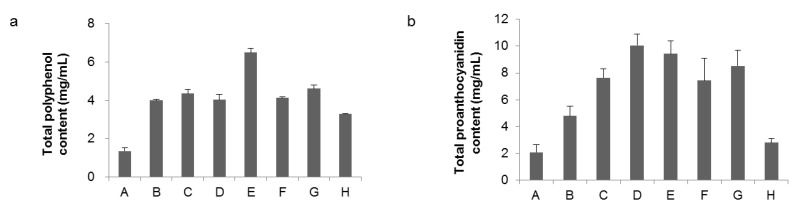
Total polyphenol and proanthocyanidin under different LED light treatment. (**a**) Total polyphenols were determined by the Folin–Ciocalteu method. Data are mg of gallic acid equivalent per 100 mg/mL DMSO solution of raspberry leaf extract; and (**b**) total proanthocyanidin was determined by a modified protein precipitation method. Data are mg of procyanidin B2 equivalent per 100 mg/mL DMSO solution of raspberry leaf extract. Error bars represent SD of the mean (*n* = 4).

Figure 2 shows the total polyphenol content measured by the Folin–Ciocalteu method and the total proanthocyanidin content using BSA adsorption. The reason why the total amount of proanthocyanidins is smaller than the total polyphenol amount is because both methods are analytical methods utilizing different properties of compounds. The numerical value of these measurement methods also varies depending on the ratio of the contained compounds and also varies depending on the standard compound used for the calibration curve. Therefore, in this study, it is used not to determine the absolute amount of the compound but to confirm the change of the relative amount under each condition. 

When grown with white LED light (conditions A and B), both total polyphenols and total proanthocyanidins decreased at 18 °C, confirming that temperature had a significant effect on polyphenol production. In the case of cultivation with only blue LED light (conditions C and D), the effect of temperature on total polyphenol amount was small, and total proanthocyanidin was increased at 22 °C. The amount of flavan-3-ol derivatives suggested that temperature effect was small under blue LED light. When the amount of blue LED light was increased (condition E), total polyphenols increased, but total proanthocyanidin level did not change. When red and blue LED lights were used at the same time (conditions F and G), there was no significant fluctuation in total polyphenols. On the other hand, total proanthocyanidins increased slightly with increasing light. While polyphenol synthesis progressed, even under zero irradiation (condition H), the total amount of proanthocyanidins decreased greatly.

### 2.3. Radical Scavenging Activity

**Figure 3:**
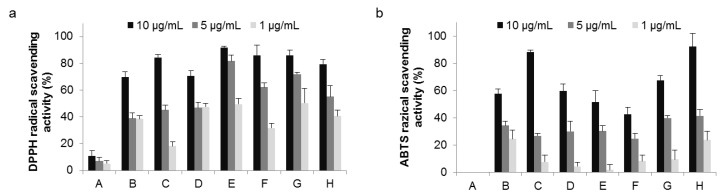
Radical scavenging activity in raspberry leaf extracts. (**a**) DPPH radical scavenging activity; one mL of 30 μM DPPH solution in EtOH was added to 1 μL of raspberry leaf extract in DMSO solution for a final concentration of 1, 5, or 10 μg/mL. Vitamin E was used as a control. Error bars represent standard deviation of the mean (*n* = 8); and (**b**) ABTS radical scavenging activity; one mL of diluted ABTS solution (absorbance at 740 nm = 0.70 ± 0.02) was added to 1 μL of raspberry leaf extract in DMSO solution for a final concentration of 1, 5, or 10 μg/mL. (+)-Catechin was used as a control. Error bars represent standard deviation of the mean (*n* = 8).

Plants rich in secondary metabolites show antioxidant activity due to their redox properties and chemical structure. The radical scavenging activity assay is commonly used as an index of antioxidant activity; here, the scavenging activity of raspberry extracts was estimated by the DPPH (a) and the ABTS radical scavenging assays (b). As shown in Figure 3, under white LED light (conditions A and B), both radical scavenging activities were reduced at 18 °C. This result correlated with total polyphenols and total proanthocyanidin. Among all conditions tested, condition E (highest blue LED light) recorded DPPH radical scavenging activity as the greatest. On the other hand, results of the ABTS radical scavenging assay were different from those of the DPPH assay, most likely due to the large difference in chemical structures and measuring activity conditions between ABTS and DPPH radicals. When structure and composition of polyphenols contained in the extract solution are different, a concomitant difference in activity is not surprising. We hypothesized that the composition of the radical scavenger contained in the raspberry leaf extract was likely changed by light treatment. Experiments are currently underway to determine both DPPH and ABTS radical scavenging assays for each compound and to identify which compounds affect activity.

### 2.4. HPLC Analysis and Identification of Polyphenols Contained in Raspberry Leaf Extracts

**Figure 4:**
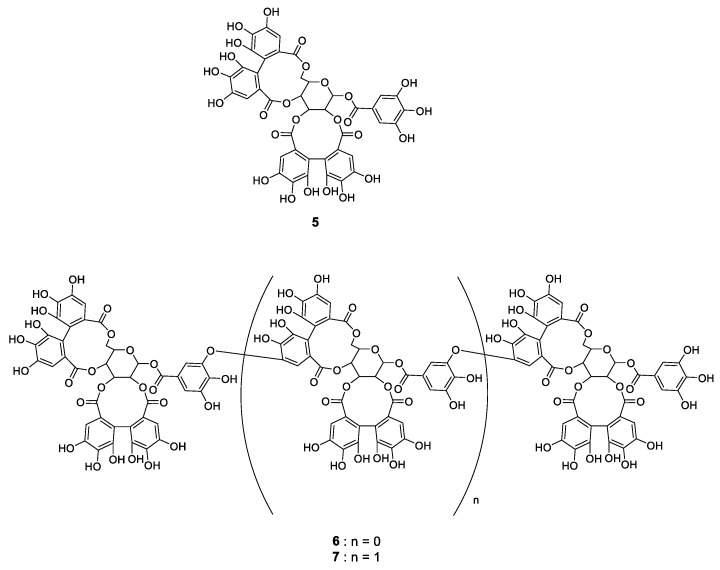
Structures of hydrolysable tannins contained in leaves of red raspberry.

**Figure 5:**
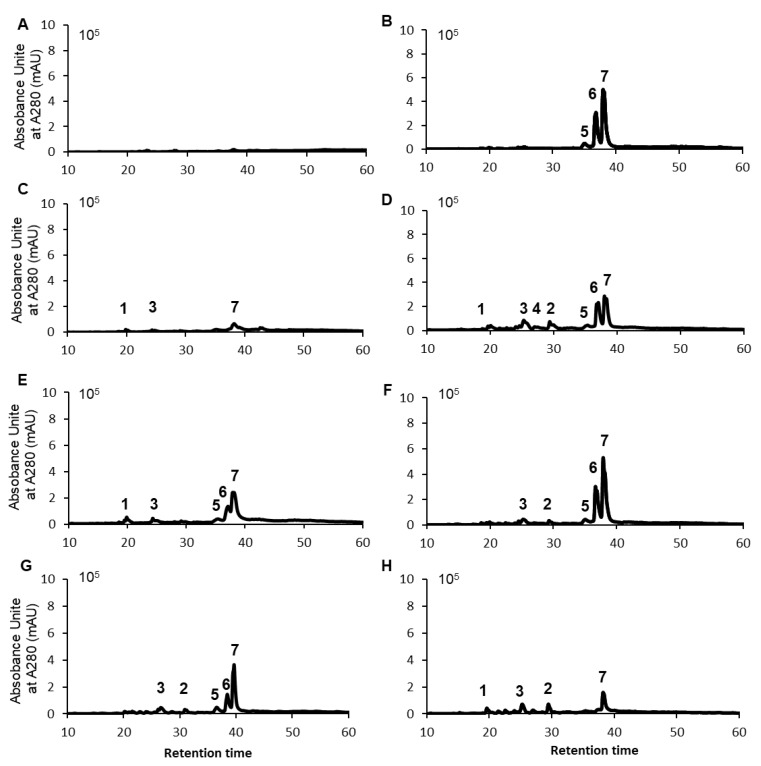
HPLC chromatograms of leaf extracts obtained from raspberry plants cultivated under condition **A** to **H**. Peak numbers are: **1**: (+)-catechin; **2**: (–)-epicatechin; **3**: procyanidin B4; **4**: flavan-3-ol trimer; **5**: 1-galloylpedunculagin; **6**: sanguiin H-6; and **7**: lambertianin C.

The results shown in Figure 3 suggested that the polyphenol composition varied with light and temperature. Thus, extracts of raspberry leaves cultivated under the range of conditions from A to H were analyzed by HPLC to identify the compounds contained and the extent to which they might change. Identification of the compounds was carried out by estimation of molecular weight by LCMS analysis and comparison with standards. Results agreed well with those of previous studies [26,27]. In addition to the flavan-3-ol derivatives shown in Figure 1, one kind of ellagitannin derivative of the hydrolysable tannins, 1-galloylpedunculagin (**5**), sanguiin H-6 (**6**), and lambertianin C (**7**), shown in Figure 4, were identified. The HPLC chromatograms for raspberry leaf extracts obtained under conditions ranging from A to H and the number of each compound on the corresponding peak are shown in Figure 5. In addition to the compounds mentioned before, quercetin, quercetin-3-*O*-glucuronide, quercetin-3-*O*-hexose, and ellagic acid were also identified; however, as they were present in minute amounts, they are not shown in the chromatogram.

Polyphenols were scarcely detected under conditions A and C, relative to which, they were much more abundant under conditions B and D, respectively. HPLC analysis confirmed that cultivation temperature greatly affected polyphenol production as well. Thus, at 22 °C, cultivation under blue light promoted the accumulation of flavan-3-ol derivatives, (compounds **1** to **4**), while hydrolysable tannins (compounds **5**~**7**) were decreased. Particularly, under condition D, the relative content of flavan-3-ol derivatives (compounds **1** to **4**) was significantly increased. Therefore, cultivation of red raspberry under blue LED light could efficiently increase flavan-3-ol derivatives. Conditions F and G also increased hydrolysable tannins as red LED light was added. Thus, irradiation with red LED light may increase the content of hydrolysable tannin (compounds **5**~**7**). On the other hand, condition H caused a high accumulation of flavan-3-ol derivatives. In this case, the amount of hydrolysable tannins was greatly reduced, while flavan-3-ol derivatives were relatively increased. These results indicate that either raspberry cultivation for a certain period under blue LED light or without light can increase leaf flavan-3-ol derivatives content.

Quantification of four raspberry flavan-3-ol derivatives based on peak areas in HPLC chromatograms is summarized in Table 2. Procyanidin B4 (**3**) increased under conditions D, E, and H. Thus, blue LED light (conditions D and E) increased flavan-3-ol derivatives, especially the dimer procyanidin B4 (**3**). These results correlated with the total amount of proanthocyanidins shown in Figure 2. As to why flavan-3-ol derivative should increase in condition H (no light), we are currently doing further experiments. Table 3 shows the amounts of flavan-3-ol derivatives **1** to **4** contained in 1.0 g of raspberry dry leaves, calculated from the amount extracted in Table 1 and the value in Table 2. When calculated on a per dried leaf weight basis, flavan-3-ol derivatives content is influenced by the dry weight extracted with methanol, and the difference is small, but in condition D, the content of procyanidin B4 (**3**) tends to be high. Among polyphenol compounds, flavan-3-ol derivatives are well known to have high functionality [31]. The possibility that the content can be increased at the level of each compound by blue LED irradiation is useful for improving the functionality of raspberry leaves.

The structure of each compound is shown in Figure 4.

### 2.5. Semi-Quantitative RT-PCR Analysis of Expression Level of the Flavan-3-ol Derivative Biosynthetic Enzyme

As the amount of polyphenols varied with light and temperature, we hypothesized that polyphenol-biosynthesis enzymes were affected by light; therefore, we measured biosynthetic enzyme-related gene expression [6,32].

**Figure 6:**
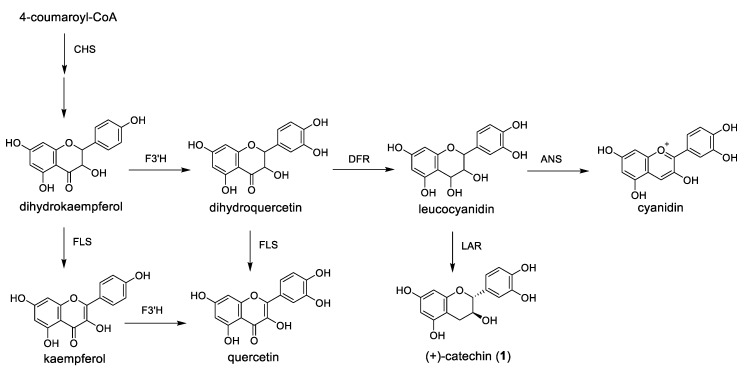
Partial schematic representation of flavonoid biosynthesis. *CHS*: chalcone synthase; *F3′H*: flavonoid 3′-hydroxylase; *FLS*: flavonol synthase; *DFR*: dihydroflavonol 4-reductase; *ANS*: anthocyanin synthase; and *LAR*: leucoanthocyanidin reductase.

Figure 6 shows some of the reactions in the biosynthesis pathway of flavan-3-ol derivatives. In order to observe an increase in the amount of procyanidin B4 present in the leaf extracts from raspberry, it is necessary to increase the synthesis of the precursor leucocyanidin. Therefore, we predicted that the expression level of *DFR* would increase, or that the expression level of *ANS* would decrease. Alternatively, it was considered that the expression level of *CHS* located upstream of the polyphenol biosynthetic pathway increased, and the total polyphenol amount might have increased.

**Figure 7:**
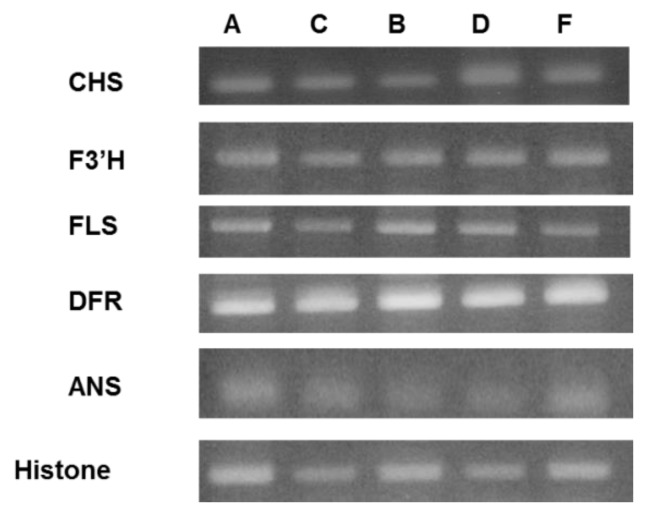
Expression level of flavan-3-ol biosynthesis enzyme encoding genes in raspberry leaves under conditions A, B, C, D, and F. Total RNA was extracted from fresh leaves after 7 days LED light irradiation treatment and used for sqRT-PCR analysis.

Figure 7 shows the semi-quantitative RT-PCR analysis of expression level of the flavan-3-ol derivative biosynthetic genes, *CHS*, *F3′H*, *FLS*, *DFR*, and *ANS*. Histone is used as a control. A, B, C, D, and F are the cultivation conditions described in Table 1. It was suggested that there is almost no difference in gene expression levels at 18 °C (conditions A, C). It is confirmed from Figure 5a that the HPLC analysis result of the extract of the leaves cultivated under condition A shows the content of the polyphenol compound is low. In other words, the change in the amount of these compounds at 18 °C was shown to be unrelated to the expression level of the enzyme gene investigated in this study. On the other hand, under the conditions B and D cultured at 22 °C, it was confirmed that only the gene expression amount of *CHS* increased under the condition D using blue LED light. That is, it was suggested that the amount of expression of *CHS* was increased by blue LED light and the flavan-3-ol derivatives were increased.

## 3. Materials and Methods

### 3.1. Reagents and Materials

HPLC grade methanol used for extraction of polyphenols and molecular biology grade dimethyl sulfoxide (DMSO) used to dissolve the crude extract were obtained from FUJI FILM Wako Pure Chemical Co. LTD. (Osaka, Japan). LC-MS grade acetonitrile and HPLC grade Formic acid were obtained from Honeywell International Inc. (Charlotte, NC, USA). All commercially available chemicals for crude extract solution were dissolved in DMSO and stored at −40 °C until used. Standard procyanidin B2 was obtained from Extrasynthese (Genay, France). Folin-Ciocalteu reagent was obtained from Merck KGaA (Darmstadt, Germany). DPPH (11-diphenyl-2-picrylhydrazyl) radical was obtained from Tokyo Chemical-Industry, Co., Ltd., Tokyo, Japan). Potassium persulfate and 2,2’-azino-bis(3-ethylbenzothiazoline-6-sulfonic acid) diammonium salt (ABTS) used to generate ABTS radical were obtained from Tokyo Chemical-Industry, Co., Ltd., (Tokyo, Japan), and FUJI FILM Wako Pure Chemical Co. Ltd., (Osaka, Japan), respectively. All other reagents and chemicals were special grade reagents, unless otherwise stated.

### 3.2. Cultivation of Raspberry

Cultivation of raspberry was carried in the plant factory unit “agri-cube” (Daiwa House Industry Co. Ltd., Osaka, Japan). Commercially available LED lamps 5000 K (Samsung Electronics Co., Ltd., Seoul, Korea) were used. Custom order LED light units that can control light quantity and irradiation wavelength (LED unit for plants, Product No. 1600435, REVOX, Inc. Kanagawa, Japan) were used for light control within the plant factory unit. The amount of PPFD (photosynthetic photon flux density) was measured by an LA-105 Light Analyzer (Nippon Medical & Chemical Instruments Co. Ltd., Osaka, Japan). The sand used for plant cultivation was purchased from the Green Garden Co. (Osaka, Japan). Himbo Top raspberry seedlings (Registration name of The Plant Variety Protection and Seed Act: RAFZAQU) used for the experiment were obtained from Tenkoen Co. (Yamagata, Japan). Roots of the raspberry seedlings were washed with water and all soil was removed. Roots were soaked in diluted mededael^®^ (MENEDAEL Co., Ltd., Osaka, Japan) for 1 min and seedlings were then transplanted into sandbags containing 3 L of sand. Sumitomo No. 2 fertilizer (Sumitomo chemical Co., Ltd., Tokyo, Japan) diluted 2000 times was applied to each sandbag at a rate of 20 mL every 8 h. A 10 cm high mesh-like bench placed under the sandbags allowed excess liquid fertilizer to drain freely. The distance from the bench to the LED was 35 cm. Plants were kept under an 18/6 h light dark regime for the duration of the experiment. Leaves were sampled seven days after combined light-temperature treatment initiation.

### 3.3. Preparation of Crude Extract Solution of Raspberry Leaves

Raspberry leaves were randomly harvested and dried at room temperature for 48 h. Dried leaves were powdered using a Speed mill MS-05 (Labonect, Osaka, Japan). To suppress heat, crushing was carried out three times for 20 s at 10 s intervals (60 s in total). Leaf samples (1.0 g) were immersed in 40 mL of HPLC grade methanol and extracted for 24 h at 25 °C. Filtration was carried out using celite as a filtration auxiliary; the filtrate was then concentrated by evaporation under reduced pressure and dried in vacuo. The obtained solid was made into a solution of 100 mg/mL with DMSO. The DMSO-solution extracts were stored at −40 °C until assayed and analyzed.

### 3.4. Total Polyphenol Analysis

Total phenolic content of the extract was determined by a modified Folin–Ciocalteu method [33,34]. Briefly, 2 μL of each crude extract (100 mg/mL of DMSO solution) was dissolved in 200 μL of Milli-Q water and mixed thoroughly with 25 μL of Folin–Ciocalteu reagent and 25 μL of 10% (*w*/*v*) sodium carbonate solution. The mixture was allowed to stand for 30 min in the dark, and absorbance was measured generationat 740 nm by a microplate reader (Multiskan FC, Thermo Fisher Scientific, Waltham, MA, USA). Total phenolic content was calculated from a calibration curve and results were expressed as mg of gallic acid equivalent per 100 mg/mL DMSO solution of raspberry leaf extract.

### 3.5. Total Proanthocyanidin Analysis

Proanthocyanidin concentration was determined by a modified protein-precipitation method described by Harbertson et al. [35] and Cáceres-Mella et al. [36]. A protein solution for proanthocyanidin precipitation was prepared by dissolving BSA in a buffer containing 200 mM acetic acid and 170 mM NaCl adjusted to pH 4.9 with NaOH, to give a final protein concentration of 1.0 mg/mL. Five μL of each raspberry leaf extract in DMSO solution was diluted with 125 μL of a buffer containing 12% ethanol (*v*/*v*) containing 5 mg/mL potassium bitartrate and adjusted to pH 3.3 with HCl. A 250 μL aliquot of protein solution was added to the diluted extract solution and incubated for 15 min at 4 °C. After centrifugation for 5 min at 15,000 rpm to pellet the proanthocyanidin protein precipitate, the pellet was washed with 50 μL of buffer containing 200 mM acetic acid and 170 mM NaCl (pH 4.9). The wash solution was poured off, 70 μL of a buffer containing 5% TAE (*v*/*v*) and 10% SDS (*w*/*v*) was added and then vortexed to dissolve the pellet completely. Ten μL of a ferric chloride reagent (10 mM FeCl_3_ in 0.01 N HCl) was immediately added to the mixture and incubated for 10 min. After incubation at room temperature, the absorbance at 520 nm was read in a microplate reader (Multiskan FC, Thermo Fisher Scientific, Waltham, MA, USA). Total proanthocyanidin content was calculated from a calibration curve and the results were expressed as the mg of procyanidin B2 equivalent per 100 mg/mL DMSO solution of raspberry leaf extract.

### 3.6. DPPH Radical Scavenging Assay

Radical scavenging activity in raspberry extracts was determined by the DPPH (1,1-diphenyl-2-picryl-hydrazyl) assay as described earlier, with some modifications [37]. Briefly, a solution of DPPH radical in EtOH (30 μM, 1.0 mL) was added to 1 μL of each synthesized compound in DMSO and incubated at 30 °C for 30 min (*n* = 6). Scavenging activity was estimated from OD readings at 515 nm obtained in a microplate reader (Multiskan FC, Thermo Fisher Scientific, CA, USA). Samples in which 1 μL of DMSO was added to 1.0 mL of EtOH, were prepared as negative controls at the same time. Absorbance readings were converted into percent radical scavenging activity as follows: [(absorbance of the control—absorbance of the sample)/absorbance of the control] × 100; vitamin E (VE) was used as standard.

### 3.7. ABTS Radical Scavenging Assay

ABTS (2,2′-azino-bis(3-ethylbenzothiazoline-6-sulfonic acid) [38] was dissolved in water to a 7 mM concentration. ABTS radical cation was produced by reacting ABTS stock solution with 2.45 mM potassium persulfate (final concentration) and allowing the mixture to stand in the dark at room temperature for 12–16 h before assay. The ABTS-radical stock solution was diluted with ethanol to an absorbance of 0.70 ± 0.02 at 740 nm. After adding 1 mL of the diluted ABTS-radical solution to 1 μL of raspberry leaf extract and mixing with Vortex, the reaction mixtures were incubated at 30 °C for 4 min. Percent scavenging activity was calculated from absorbance readings at 740 nm as follows: [(absorbance of the control—absorbance of the sample)/absorbance of the control] × 100; (+)-Catechin was used as standard.

### 3.8. LC-MS Analysis Conditions

LC-MS was performed using a Shimadzu LCMS-2020 system equipped with a DGU-20A_3R_ degas unit, LC-20A binary pump, SIL-20AC auto sampler, SPD-M20A diode array detector, CTO-20AC column oven and CBM-20A communications bus module connected to a LC work station (Shimadzu Corporation, Kyoto, Japan). A Wakopac^®^ MS-5C18GT column (φ 150 mm × 2.0 mm, 5 μm, FUJIFILM Wako Pure Chemical Corporation, Osaka, Japan) was selected. Briefly, 0.05% (*v*/*v*) formic acid was mobile phase A and acetonitrile was mobile phase B. Analyses of raspberry extracts were achieved using a linear gradient from 0%–35% B over 0–90 min; 35%–100% B over 90–95 min; 100% B, over 95–100 min; 0% B, over 100–110 min. The flow rate was set at 0.2 mL/min. The injection volume was 10 μL and the temperature of the column oven was maintained at 40 °C.

Each DMSO-solution sample extract was diluted with 20% aqueous acetonitrile solution and filtered through a pre-column Sep-Pak Cartridge (Waters Corp., Milford, MA, USA) to remove lipophilic compounds.

A Shimadzu 2020 Quadrupole Mass Spectrometer (Shimadzu Corporation, Kyoto, Japan) equipped with a positive/negative ESI source was used as a detector. The mass spectrometer was operated in the negative selected-ion-monitoring (SIM) with capillary voltage at 1.2 V for phenolic compounds identification, and in a positive SIM for betanin. Conditions for MS analysis were designed as follows: Spray voltage was −3.5 V, dissolving line temperature was 250 °C, nebulizer gas flow was 1.5 L/min, the heat block was set at 200 °C, drying gas flow was 12.00 and 15.00 for phenolics and betanin, respectively; finally, detector voltage was 1.2 V.

Identification of flavan-3-ol derivatives was carried out by comparison of retention time and mass spectrum to standard samples. Commercially available catechins and epicatechin were used for identification. Procyanidin B4 was synthesized by our group. The flavan 3-ol trimer was determined to be a trimer based on similarity of MS spectra with procyanidins C1, C2, C4, and other trimers. However, the structure was not consistent with the procyanidin trimer that we synthesized, and which has been confirmed by organic synthesis. Quantitation of each compound amount was calculated by preparing a calibration curve for each compound.

### 3.9. Semi-Quantitative RT-PCR Analysis of Flavan-3-ol Derivative Biosynthetic-Enzyme-Related Gene Expression

Total RNA was extracted from raspberry leaves using a RNeasy Plant Mini Kit (Qiagen, Valencia, CA, USA) for semi-quantitative reverse-transcription polymerase chain reaction (RT-PCR). After treatment of RNA samples with DNase using a DNA free kit (Ambion; Applied Biosystems Japan, Tokyo, Japan), cDNAs were synthesized using SuperScript III reverse transcriptase (Invitrogen, Carlsbad, CA, USA), followed by incubation with RNase H (Invitrogen). RT-PCR was performed using GoTaq Green Master Mix (Promega, Madison, WI, USA) in a T100TM Thermal Cycler (Bio-Rad Laboratories, Hercules, CA, USA) with the gene-specific primer sets shown in Table 4 [6,32,39]. Reaction conditions were as follows: CHS: After heating 95 °C for 3 min, PCR was performed for 40 cycles (95 °C 30 s, 50 °C 30 s, and 72 °C 22 s); ANS: after heating 95 °C for 2 min, PCR was performed for 30 cycles (95 °C 1 min, 55 °C 20 s, and 72 °C 10 s); FLS, DFR and F3′H: after heating 95 °C for 5 min, PCR was performed for 30 cycles (95 °C 30 s, 50 °C 30 s, and 72 °C 45 s); Histone: after heating 95 °C for 3 min, PCR was performed for 29 cycles (95 °C 30 s, 55 °C 30 s, and 72 °C 25 s).

## 4. Conclusions

We investigated to clarify the effect of light and temperature on flavan-3-ol content in raspberry leaves. When raspberries were cultivated in plant factory unit and light intensity, wavelength and temperature were changed, the amount of total polyphenols increased under blue light. HPLC analysis performed with the determination of flavan-3-ol derivatives (+)-catechin, (−)-epicatechin, procyanidin B4, flavan-3-ol trimer, and increase under blue light confirmed. Semi-quantitative RT-PCR showed a correlation between chalcone synthase (CHS) gene expression and the amount of compound measured in the leaves.

## Figures and Tables

**Table 1:** Cultivation conditions and amount of extract obtained from raspberry leaves.

-	A ^1^	B ^1^	C	D	E	F	G	H
PFD-B (Blue) (μmol m^−2^ s^−1^)	7.23	7.23	30.24	30.24	40.09	3.86	16.54	-
PFD-R (Red) (μmol m^−2^ s^−1^)	8.33	8.33	-	-	-	4.43	27.87	-
Temperature (°C)	18	22	18	22	22	22	22	22
Extract (g) ^2^	0.37	0.30	0.32	0.28	0.25	0.30	0.33	0.30

^1^ In conditions A and B, cultivation was carried out using commercially available white LED. ^2^ Dry weight of the extract obtained from 1.0 g samples of dried leaves cultivated under each condition.

**Table 2:** Flavan-3-ol derivatives (mg/100 mg of DW) in raspberry leaf extracts.

-	A	B	C	D	E	F	G	H
(+)-catechin (**1**)	0.50	0.44	0.45	0.48	0.60	0.46	0.45	0.52
(–)-epicatechin (**2**)	0.10	0.09	0.06	0.10	0.11	0.09	0.11	0.11
procyanidin B4 (**3**)	0.59	0.58	0.48	1.25	1.00	0.79	0.87	1.35
flavan-3-ol trimer (**4**)	0.34	0.32	0.30	0.38	0.38	0.34	0.37	0.39

Data are means of three technical replications of each calculation based on HPLC measurements.

**Table 3:** Amount of each flavan-3-ol derivative (mg/g DW) in raspberry leaves.

-	A	B	C	D	E	F	G	H
(+)-catechin (**1**)	1.85	1.32	1.44	1.34	1.5	1.38	1.49	1.56
(–)-epicatechin (**2**)	0.37	0.27	0.19	0.28	0.28	0.27	0.36	0.33
procyanidin B4 (**3**)	2.18	1.74	1.54	3.5	2.5	2.37	2.87	4.05
flavan-3-ol trimer (**4**)	1.26	0.96	0.96	1.06	0.95	1.02	1.22	1.17

**Table 4:** The sequences of the primer used for RT-PCR experiments.

Gene	Accession No. of Sequence Used for Primer Design	Primer Sequence
CHS	AF292367	**F:** AACCCTTGTTTCTTCGTACCATTA
		**R:** GATGGGTAGCTAGTACTTACACAT
ANS	KX950789.1	**F:** ATC GTC ATG CAC ATA GGC GAC ACC
		**R:** CCT TGG GCG GCT CAG AGA AAA
FLS	GT029981	**F:** AGG TGA ACA GGT GGA GTT GG
		**R:** TGA AGA CCA TCA TCG AAT GC
DFR	GT029979	**F:** ATG CGA AAC AAC TTG CAT TT
		**R:** GCT ACG ATT CAC GAC ATT GC
F3′H	GT029980	**F:** TGA TGA AGC TTT ATA AGC ATG TGA GG
		**R:** GGG TCC ACT CTC TTG GTG AA
Histone	AF304365.1	**F:** CAA GGA AGC AAT TGG CTA CCA AGG
		**R:** AGT TGG ATA TCC TTG GGC ATA ATA
